# Effects of the Vitamin D3 on Alleviating the Oxidative Stress Induced by Diquat in Wenchang Chickens

**DOI:** 10.3390/ani13040711

**Published:** 2023-02-17

**Authors:** Keyi Nong, Youming Liu, Xin Fang, Xinyun Qin, Zhineng Liu, Haiwen Zhang

**Affiliations:** College of Animal Science and Technology, Hainan University, Haikou 570228, China

**Keywords:** Vitamin D_3_, antioxidant, broiler, intestinal health, biochemical blood indexed, production performance

## Abstract

**Simple Summary:**

Our study showed that a Vitamin D_3_ (VD_3_) addition in Wenchang chickens’ diet could improve the adverse effects of diquat (DQ) on the performance and oxidation status of broilers, recover the secretion of inflammatory-related factors, and the content of serum biochemical indicators associated with intestine, liver, and kidney injury, and increase antioxidant enzyme activity. In addition, the liver and intestinal morphology were protected after supplementing VD_3_. This is a research basis for the application of VD_3_ as a feed additive in poultry against oxidative stress.

**Abstract:**

Vitamin D_3_ (VD_3_) is an indispensable micronutrient in livestock and poultry feed. Its function in antioxidant stress has been reported. We investigate whether the addition of different concentrations of VD_3_ to the diet affects the production performance, slaughter performance, meat quality, organ index, and gut injury on the diquat (DQ)-induced model of oxidative stress in Wenchang chickens. Four hundred and eighty one-day-old chickens were randomly divided into six groups: control (basal diet), 4000 VD (basal diet + VD_3_ 4000 IU per kg feed intake), 1000 VD+DI (DQ, basal diet + VD_3_ 1000 IU per kg feed intake), 2000 VD+DI (DQ, basal diet + VD_3_ 2000 IU per kg feed intake), and 4000 VD+DI (DQ, basal diet + VD_3_ 4000 IU per kg feed intake). The results showed that the addition of VD_3_ to the diet promoted DQ-induced weight loss and reduced ADFI, slaughter rate, splenic index, and pH after 1 h and 24 h in the leg muscles. VD_3_ decreased the increase in content of interleukin-1β (IL-1β), interleukin-6 (IL-6), and tumor necrosis factor-α (TNF-α) among proinflammatory cytokines (*p <* 0.05) and increased the reduction in anti-inflammatory cytokines content of interleukin-10 (IL-10) (*p <* 0.05) induced by DQ. In addition, liver and kidney injury biomarkers and the intestinal permeability index in serum were disordered after treatment with DQ (*p <* 0.05). VD_3_ perfected the increase of D-lactic acid (D-LA), diamine oxidase (DAO), total cholesterol (T-CHO), creatinine (CR), blood urea nitrogen (BUN), triglycerides (TG), low-density lipoprotein cholesterol (LDL-C) content, aspartate transaminase (AST), alanine transaminase (ALT), and lactate dehydrogenase (LDH) activity (*p <* 0.05); it increased the decrease of albumin (ALB) content (*p <* 0.05). Meanwhile, VD_3_ regulated the intestinal morphology and intestinal barrier. Moreover, DQ induced a decrease in total antioxidant capacity and antioxidant enzyme activity in the serum, liver, and jejunum (*p <* 0.05), and an increase in malonaldehyde (MDA) content (*p <* 0.05). However, the addition of different levels of VD_3_ could alleviate the above phenomenon of oxidative stress in Wenchang chickens to different degrees. Thus, this research suggested that the addition of VD_3_ can relieve the DQ-induced oxidative stress of Wenchang chickens, and the level of VD_3_ acquisition is positively correlated with the remission effect.

## 1. Introduction

With the rapid development of modern agriculture, oxidative stress is a widespread phenomenon in poultry production with the rapid growth of modern agriculture [1]. Many reasons can cause oxidative stress in poultry production, including environmental and temperature changes, transportation, intensive farming, and nutrition [2]. Oxidative stress representing Oxidative stress representing the equilibrium relationship between the production of reactive oxygen species (ROS) and the capacity of antioxidant scavenging has broken [3]. Oxidative stress can lead to serious broiler injuries, such as a decrease in growth performance, the breakdown of antioxidant defenses, organic damage [4], and an impaired host immune response [5]. Among them is oxidative stress-induced liver and kidney damage, according to a previous report [6,7]. The important role of the liver and kidney in the biosynthesis and metabolism of the body determines that the damage to liver and kidney tissue caused by ROS is worthy of attention [8]. At the same time, oxidative stress is associated with an inflammatory response, and immune stress induces intestinal inflammation, which causes intestinal mucosal damage and ultimately intestinal epithelial dysfunction [9,10]. So, we need to find a beneficial nutrition strategy to face the impact of oxidative stress in poultry breeding.

As an oxidative stressor, diquat (DQ), 1,1′-ethylene-2,2′-bipyridine dibromide, is known as a non-specificity defoliant [11]. It can directly catalyze molecular oxygen to generate superoxide anion and H_2_O_2_, trigger lipid peroxidation, and produce a mass of free radicals, significantly increasing the ROS in the gut [12,13]. It will bring virulence to the lungs, kidneys, and liver. The toxic effect mainly comes from the ROS [14]. Studies indicate that the production of the ROS can induce oxidative stress in the process of the oxidation-reduction cycle. Chen et al. (2020) found that the broiler MDA content significantly increases, and superoxide dismutase (SOD) and glutathione peroxidase (GHS-Px) activity significantly decreases in blood and liver after injecting DQ (20 mg/kg BW) [1]. Another study reported that DQ leads to the decline of growth performance, the degradation of liver function, and oxidative status [3]. In addition, DQ also has the characteristics of being of a low dosage, manageable to control, and takes effect rapidly. DQ is good at inducing oxidative stress in animals which affects growth performance and nutrition metabolism [15]. Therefore, it is considered reasonable to use DQ as an inducer of oxidative stress.

Vitamin D_3_ (VD_3_) is an existing form of Vitamin D (VD), a steroid hormone [16]. It is ingested in animal feeds. By shining ultraviolet light on 7-dehydrocholesterol in the skin, it becomes the 25-hydroxyvitamin D_3_ (25-OH-D_3_), one of the storage forms of VD_3_, after entering the liver through blood, and then it is the active form 1α, 25-dihydroxyvitamin D_3_ (1α, 25-(OH)_2_-D_3_) in the kidney or other organs [17]. Animals mainly exert physiological functions through 1α, 25-(OH)_2_-D_3_ [18]. In livestock and poultry feed, VD_3_ has become an indispensable feed additive because of its function in regulating calcium and phosphorus balance and skeletal muscle growth [19]. As there are more and more studies of VD_3_’s effects on humans and animals, the findings of the Vitamin D receptor (VDR) suggest that VD’s effects on cells do more than that. VDR has been detected in many tissues, including adipose tissue, skin, immune system cells, and the placenta. VD was shown to be specific in the endocrine and digestive systems, especially in the small intestine, as large numbers of VDRs were found in these regions [20]. Mainly, VDR is expressed in all immune cells, some of which are also able to synthesize or respond to biologically active metabolites, allowing for autocrine and paracrine effects of VD_3_ [21]. VD deficiency is often an indirect cause of the multi-stage onset of the disease, and VD supplementation is essential for the prevention of certain diseases [22]. VD_3_ has been proven to have anti-inflammatory and antioxidant effects [23]. According to the previous report, a lack of VD_3_ decreased egg production and quality, resulting in increased serum calcitonin and estradiol levels, and elevated levels of inflammation-related markers (IL-6, IL-10, myeloperoxidase, NF-κB, inducible nitric oxide synthase) and LPS treatment induced IgM level in serum and CD8 + T cellular percentage increase, not only protecting chicken eggs from damage but immune stress is relieved through supplementary VD_3_ [24]. In mouse models, 25-OH-D_3_ inhibits inflammatory factors’ production and viral replication has been shown [25]. Moreover, Wang et al. (2021) researched that adding 25-OH-D_3_ to the diet improved the gut barrier, antioxidant, and intestinal microbial composition of laying hens [26]. In addition, the defensins are produced at epithelial surfaces, In addition, the defensins are produced at epithelial surfaces, benefiting the host immune, associated with intestinal inflammation [27]. VD_3_ induces the expression of chicken β-defensin genes in embryonic intestinal epithelial cells and peripheral blood mononuclear cells [28]. It acts as an immunomodulator to improve the congenital immunity response of chickens [29]. Our previous study found that oral gavage of VD_3_ daily can improve the abnormal change in weight gain, immune organ index, antioxidant and inflammatory levels, and intestinal injury induced by DQ [30].

Given the previous studies, VD_3_ is a feed additive with broad application prospects. Though there are some reports about the function of VD_3_ in anti-infection and immunity, the antioxidant stress was few. Wenchang Chicken is a local brand of Hainan tropical characteristics and efficient agriculture. It is known for its meat quality. It was always caged to enhance the flavor of the meat and ensure the scale of breeding. In the rearing process of Wenchang chickens, exogenous pathogenic microorganisms’ infection, high temperature and humidity environment, and dietary nutrition will cause oxidative stress and affect the production performance. However, the effects of VD_3_ supplemented to the diet of Wenchang chickens to cope with the oxidative stress induced by DQ remain unknown. Thus, the different concentrations of VD_3_ alleviating the oxidative stress induced by DQ on Wenchang chickens was explored in this study.

## 2. Materials and Methods

### 2.1. Experimental Animals

**Figure 1:**
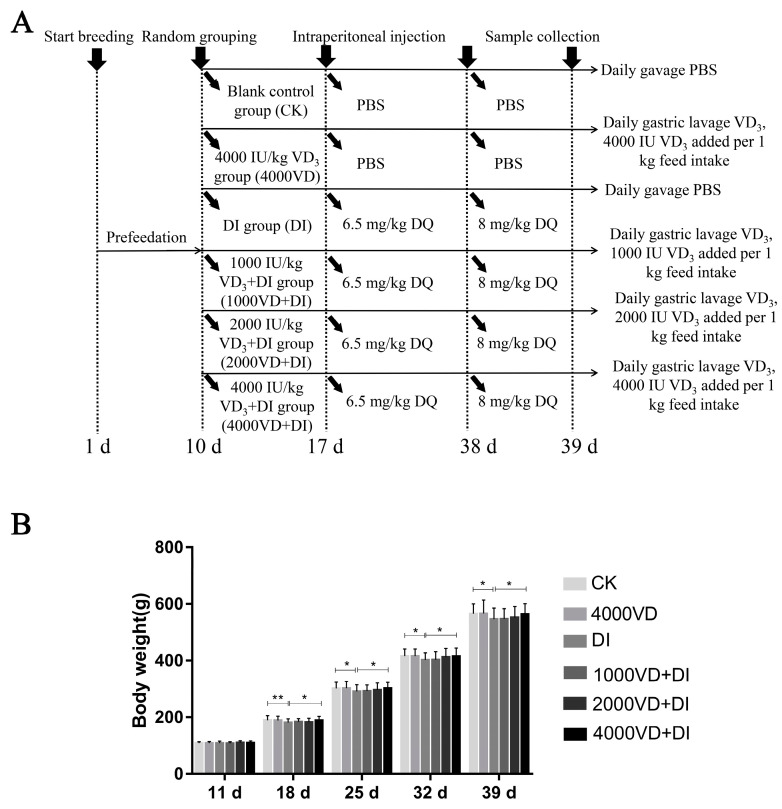
(**A**) Experimental treatment diagram of each group and (**B**) the effect of different supplemental levels of VD_3_ on the body weight of the Wenchang chicken oxidative stress model. (Statistical analysis of the data was performed using the Duncan method to analyze the significance of differences between samples by IBM SPSS software, * *p <* 0.05, ** *p <* 0.01).

Four hundred and eighty 1-day-old healthy Wenchang chickens were purchased from a hatchery (Tanniu Wenchang Chicken Hatchery, Haikou, Hainan, China) and raised for a 10-d adaptation period. The chickens were randomly divided into six treatments of ten replicates, each with eight birds per replicate (all females). As shown in the abridged general view (Figure 1A): the control group (CK), VD_3_ (V8070, Beijing Solarbio Science & Technology Co., Ltd., Beijing, China) the addition alone group (4000VD), the DQ-induced (D101258, Aladdin Reagent Co., Ltd., Shanghai, China) model group (DI), and the VD_3_ addition + DQ group (1000VD+DI, 2000VD+DI, and 4000VD+DI). Firstly, the broilers in the 4000VD, 4000VD+DI, 2000VD+DI, and 1000VD+DI groups, according to their feed intake, were, respectively, administrated the 4000 IU/kg, 4000 IU/kg, 2000 IU/kg, and 1000 IU/kg 200 μL VD_3_ solutions (which was dissolved in 0.2% ethanol and diluted with sterile PBS) daily through oral gavage. The other groups received 200 μL PBS (P1010, Beijing Solarbio Science & Technology Co., Ltd., Beijing, China). On days 17 and 38, the DI, 4000VD+DI, 2000VD+DI, and 1000VD+DI groups were administered a 6.5 and 8 mg DQ per kg BW (suspended in 200 μL PBS) intraperitoneal injection. The CK group and 4000VD group were injected with 200 μL PBS. The nutritional needs in the brooding period (days 1 to 21) and growing period (days 22 to 38) in the basal diets were as recommended by the NRC (1994) and “chicken breeding standard: NY/T33-2004” (Table 1).

The chickens were placed in an environmentally controlled experimental hencoop (150 cm long, 150 cm wide, and 200 cm high) with a plastic-wire floor. The chickens were held at a brooding temperature of 34 ± 1 °C for the first week, and then it was gradually decreased by 2 to 3 °C per week until it was maintained at 22 °C. The chickens were given fresh water and the above diets with a 24 h constant light schedule.

### 2.2. Growth Performance

The initial weight and final weight were recorded. The BW of each chicken was weighed before oral administration every day. Meanwhile, the feed intake of broilers in each group was recorded daily. The end of the test, average daily gain (ADG), average daily feed intake (ADFI), and feed/gain (F/G) were calculated.

1. ADFI = the total feed intake/(trial days × number of test animals)
2. ADG = (final weight − initial weight)/measured days
3. F/G = total feed consumption/(final weight − initial weight)

### 2.3. Sample Collection and Preparation

At 39 d, bloods were stochastically collected from 20 broilers in each treatment (2 birds per replicate) by puncturing the main vein of the wing after weighing. Non-anticoagulated blood samples stood still at 25 °C for 1 h. The serum was transferred to a 1.5 mL centrifuge tube after isolating by centrifugation for 10 min (4 °C, 3000 rpm), respectively. Then the broilers were anesthetized with sodium pentobarbital and necropsied. The new samples, including intestinal tissue, liver, and kidney, were rapidly removed artificially from carcasses in a low-temperature environment (ice-cold surface). In a short time, a part of the above tissue samples was cut and dipped in 4% paraformaldehyde to make the paraffin section. The samples of jejunum, liver, and serum were used for further research.

### 2.4. Determination of the Slaughter Performance and Organ Index

During the slaughtering process, live weight, carcass weight, and evisceration weight of the chickens were weighed and calculated. Then, the excising and weighing of the liver, kidney, spleen, and bursa of Fabricius. The organ index (organ weight (mg)/broiler weight g)) was calculated.

### 2.5. Meat Quality

Both sides of the pectoral and leg muscles were collected for analysis:

1. Three different points (a depth of 2.5 cm) of the pectoral or leg muscles were measured using a pH meter (SIN-PH160, Hangzhou Lianji Automation Technology Co., Ltd., Hangzhou, China) at 1 h (pH 1 h) and 24 h (pH 24 h) postmortem.

2. Approximately a 4 g meat sample was placed in a plastic bag in a 75 °C water bath, cooked for 0.5 h and then blotted dry and weighed. The cooking loss was calculated ((raw weight − cooked weight)/raw weight × 100%).

3. Approximately a 4 g meat sample in a transparent plastic bag was stored at 4 °C for 1 d, and then was weighed. Drip loss values were calculated ((raw weight − stored weight)/raw weight × 100%).

### 2.6. Detection of the Biomarker in the Serum

The activities of DAO (A088-2-1), ALT (C009-2-1), AST (C010-2-1), and LDH (A020-2-2), and the content of D-LA (H263-1-2), TG (A110-1-1), LDL-C (A113-1-1), T-CHO (A111-1-1), BUN (C013-2-1), CR (C011-2-1), and ALB (A028-2-1) in serum were measured with corresponding assay kits (Nanjing Jiancheng Bioengineering Institute, Nanjing, China) based on the specification.

### 2.7. Determination of Antioxidative Enzymes in the Serum, Liver, and Jejunum

The handling method of the liver and jejunum sample was the same as mentioned. The total antioxidant capacity (T-AOC, A015-2-1), the activities of SOD (A001-3-2), GSH-Px (A005-1-2), and catalase (CAT, A007-1-1), and the content of MDA (A003-1-2) in the serum, liver, and jejunum were measured with corresponding assay kits (Nanjing Jiancheng Bioengineering Institute, Nanjing, China). Protein normalization was used for comparison between samples. The BCA kit was used to determine concentration (A045-4-2).

### 2.8. Detection of the Inflammatory Cytokines in Serum

The concentrations of IL-1β (EK0394), IL-6 (EK0411), TNF-α (EK0527), and IL-10 (EK0417) in serum were determined through an ELISA kit (Boster Biological Technology Co., Ltd., Wuhan, China).

### 2.9. Evaluation of Morphology in the Duodenum, Jejunum, Ileum, and Liver Tissues

The midpiece of the duodenum, jejunum, and ileum and the partial liver were taken to conduct hematoxylin and eosin (H&E) staining (G1003, Wuhan Service bio–Biotechnology Co., Ltd., Wuhan, China). Briefly, the samples from the 4% paraformaldehyde solution (G1101, Wuhan Service bio–Biotechnology Co., Ltd., Wuhan, China) were immersed in paraffin and cut into slices. Then, they were stained with hematoxylin and eosin. The sections were observed, under different magnifications, using a Leica NEWDM 4500BR microscope (Leica, Frankfurt, Germany). Optec OPTPro x64 (Version 3.7.13522.20181209, Chongqing, China) software was used to collect images; villus height (VH) and crypt depth (CD) were measured.

### 2.10. Determination of the Apoptotic Level of the Jejunum Epithelium

To evaluate the apoptosis degree of the jejunal epithelium, the same part of the jejunum was deparaffinized, and then xylene (10023418, Sinopharm Chemical Reagent Co., Ltd., Shanghai, China) was applied to increase the transparency of slices. The slices were stained with a TdT-mediated dUTP Nick-End Labeling staining (TUNEL) kit (G1507, Wuhan Service bio–Biotechnology Co., Ltd., Wuhan, China). More specifically, terminal deoxyribonucleotidyl transferase and 2′-deoxyuridine 5′-triphosphate were mixed (1:9) and incubated with the slices (37 °C, 60 min). The endogenous peroxidase was blocked, and the slices were dried naturally. The slices were then covered with converter peroxidase and incubated for another (37 °C) 30 min. The slices were added to Diaminobenzidine (G1212, Wuhan Service bio–Biotechnology Co., Ltd., Wuhan, China), and coloration was stopped with distilled water. Next, hematoxylin as a counterstain was used to stain the cell nucleus. The slices were dehydrated and mounted, finally.

### 2.11. Detection of the Expression Level of Polysaccharides in the Jejunum Tissue

Similarly, the same part of the jejunum was used for the analysis of the expression level of polysaccharides in the jejunum tissue. In short, the samples were deparaffinized, and the section transparency was increase by using xylene. The slices were stained with a periodic acid-Schiff staining (PAS) solution (G1008, Wuhan Service bio–Biotechnology Co., Ltd., Wuhan, China) and washed with water. The slices were dehydrated and mounted.

### 2.12. Statistical Analysis

All data were collated by WPS Excel (Version 2022, Beijing, China). One-way analysis of variance was undertaken followed by IBM SPSS Statistics (Version 23, New York, NY, USA) software. The significance of differences between every two groups was carried out by Duncan’s multiple group comparisons. *p* ≤ 0.05 or *p* ≤ 0.01 means that the results are statistically significant. Results were expressed as mean ± SEM. The figures were created by GraphPad Prism (Version 7.0, San Diego, CA, USA). The slices of TUNEL and PAS were observed with a microscope, and the images were collected with Optec OPTPro x64 software.

## 3. Results

### 3.1. Effect of VD_3_ Addition on Growth Performance

As shown in Figure 1B, there was no significant difference in the weight of the chickens in each group when they were 11 days old (*p >* 0.05). After an intraperitoneal injection of DQ at 17 days of age, the age of 18 (*p <* 0.01), 25 (*p <* 0.05), 32 (*p <* 0.05), and 39 (*p <* 0.05) days showed that the weight of the CK group and the 4000VD group had a significant difference compared with the DI group, and the 4000VD+DI group showed a significant difference compared with the DI group (*p <* 0.05). The final weight of the control group and 4000VD groups were lower than that of the DI group (*p <* 0.05), and that in the 4000VD+DI group was higher than the DI group (*p <* 0.05), even recorded to the same level as the control group and 4000VD group (Table 2). The ADFI of the 4000VD+DI group was higher than that of the DI group (*p <* 0.05). However, during the trial, the ADG and F/G of each group of chickens did not show a significant difference (*p >* 0.05) (Table 2). The result indicated that VD_3_ had no effect on the growth performance of chickens but improved the effect of weight change caused by DQ.

### 3.2. Effect of VD_3_ Addition on Slaughter Performance and Organ Index

As shown in Table 3, oxidative stress reduced the dressing percentage of chickens; the DI group was significantly lower than that in the CK and 4000VD groups (*p <* 0.05). As the concentration of the VD_3_ addition increased, the decreasing dressing percentage caused by DQ gradually recovered. In Table 4, the spleen index of the CK and 4000VD groups was lower than that of the DI group (*p <* 0.05). With the addition of VD_3_, the spleen index gradually returned to normal levels. There was no significant difference between the 4000 VD+DI and CK groups (*p >* 0.05). The above results showed that the addition of VD_3_ improved the dressing percentage and the spleen development of Wenchang chickens after DQ was damaged.

### 3.3. Effect of VD_3_ Addition on Meat Quality

There is no difference between the CK group and the DI group in the meat quality pH index of the pectoral (*p >* 0.05) (Table 5). As shown in Table 6, compared with the CK group, the pHs at 1 h and the 24 h of the leg muscles of the DI group were significantly reduced (*p <* 0.05). After the VD_3_ addition, there was no significant difference between the 4000 VD+DI and CK groups (*p >* 0.05). The result indicated that the VD_3_ addition improves the pH of leg muscles.

### 3.4. Effect of VD_3_ Addition on the Intestines

#### 3.4.1. Effect of VD_3_ Addition on the Secretion of Inflammatory Cytokines in Serum

**Figure 2:**
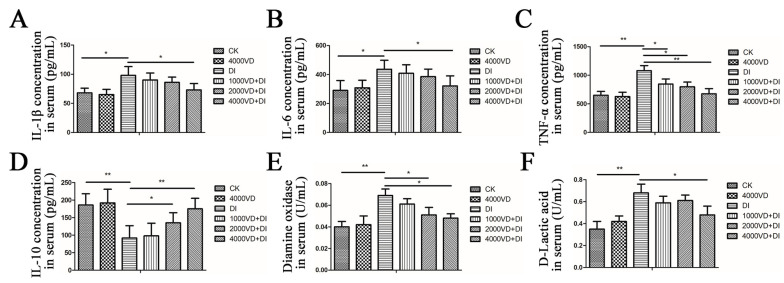
Effects of different supplement levels of VD_3_ on serum intestinal injury markers and inflammatory cytokines in the Wenchang chicken oxidative stress model. (**A**) The IL-1β concentration of each group, (**B**) IL-6 concentration of each group, (**C**) TNF-α concentration of each group, (**D**) IL-10 concentration of each group, (**E**) DAO activity in the serum of each group, (**F**) D-LA content in the serum of each group. (Statistical analysis of the data was performed using the Duncan method to analyze the significance of differences between samples by IBM SPSS software, * *p <* 0.05, ** *p <* 0.01).

As shown in Figure 2A–D, oxidative stress causes an increase in serum concentrations of IL-1β, IL-6, and TNF-α, and a decrease in IL-10 concentration. Compared with the CK group and the 4000VD group, IL-1β, and IL-6 have significantly increased in the DI group (*p <* 0.05). IL-10 and TNF-α have highly significant differences between the DI group and the CK group (*p <* 0.01). With the increase of the VD_3_ addition concentration, the abnormal levels of inflammatory factors in serum caused by DQ gradually returned to normal, and there was no significant difference between the 4000VD+DI group and the CK group (*p >* 0.05). These results indicate that the VD_3_ supplement could improve the disorder of inflammatory factors induced by DQ.

#### 3.4.2. Determination of Biomarkers of Intestinal Injury in Serum

Oxidative stress causes a significant increase in the serum’s D-LA content and DAO enzyme activity. The D-LA content and DAO enzyme activity of the DI group significantly differed from the CK group and the 4000VD group (*p <* 0.01). With the gradual increase of the VD_3_ addition, it gradually decreased (Figure 2E,F). The result indicates that the intestinal injury induced by DQ was protected by the VD_3_ intervention.

#### 3.4.3. Effect of VD_3_ Addition on Intestinal Tissue Morphology

**Figure 3:**
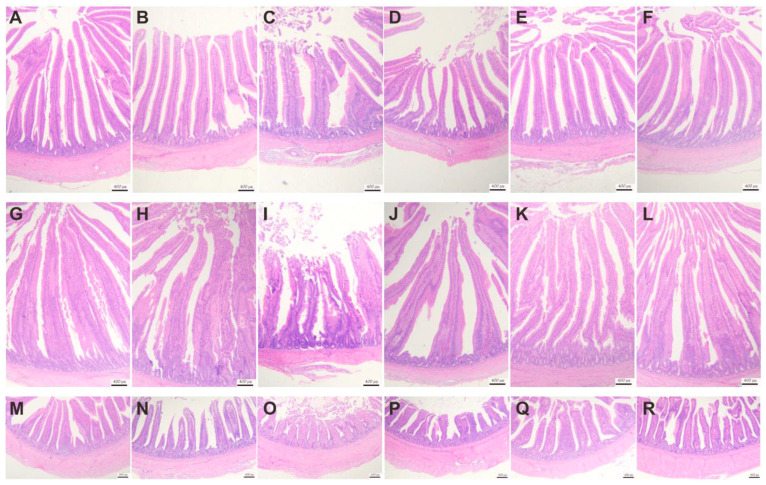
Effects of different levels of VD_3_ supplementation on the intestinal morphology of the Wenchang chicken oxidative stress model (40×). (**A**–**F**): jejunum morphology. (**A**) CK group, (**B**) 4000VD group, (**C**) DI group, (**D**) 1000VD+DI group, (**E**) 2000VD+DI group, (**F**) 4000VD+DI group. (**G**–**L**): duodenal morphology. (**G**) CK group, (**H**) 4000VD group, (**I**) DI group, (**J**) 1000VD+DI group, (**K**) 2000VD+DI group, (**L**) 4000VD+DI group. (**M**–**R**): ileum morphology. (**M**) CK group, (**N**) 4000VD group, (**O**) DI group, (**P**) 1000VD+DI group, (**Q**) 2000VD+DI group, (**R**) 4000VD+DI group.

**Figure 4:**
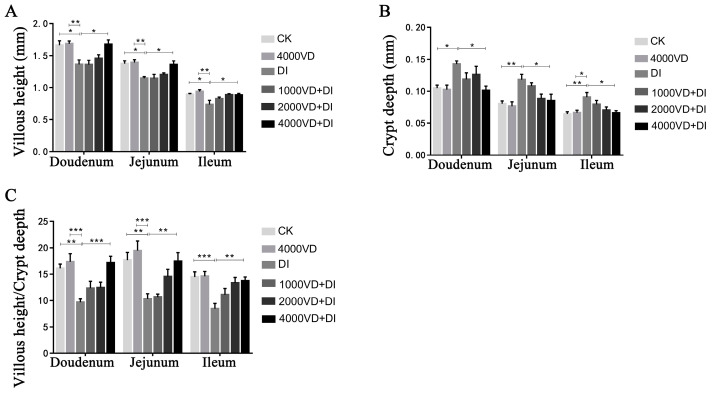
The villous height, crypt depth, and villous height/crypt depth. (**A**) the villous height of each intestinal segment. (**B**) the crypt depth of each intestinal segment. (**C**) the villous height/ crypt depth of each intestinal segment. (Statistical analysis of the data was performed using the Duncan method to analyze the significance of differences between samples by IBM SPSS software, * *p <* 0.05, ** *p <* 0.01, *** *p <* 0.001).

The intestinal morphology of the duodenum, jejunum, and ileum is shown in Figure 3, and oxidative stress causes damage to the mucosal structures of the duodenum, jejunum, and ileum. In the DI group, there was a detachment of epithelial cells of intestinal villi and pathological edema of the submucosal layer of the jejunum. By adding different concentrations of VD_3_, intestinal damage is gradually reversed. As shown in Figure 4, the VH of the DQ group was significantly lower than that of the control group, while the VD_3_ treatment group changed this situation. In contrast, the CD was significantly higher in the DI group than in the CK group, while this increase was mitigated in the VD_3_ treatment group. There were the same changes in the duodenum, jejunum, and ileum. The result indicates that VD_3_ could maintain the normal shape of intestinal villi (*p <* 0.05 or *p <* 0.01).

#### 3.4.4. Effect of VD_3_ Addition on the Level of Apoptosis of Epithelial Cells in Jejunal Tissue

**Figure 5:**
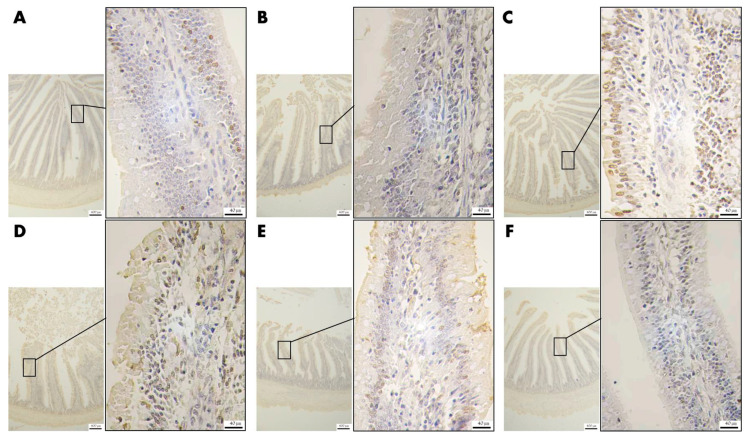
Effects of different levels of VD_3_ supplementation on apoptosis of jejunal epithelial cells in the Wenchang chicken oxidative stress model (40× and 400×). (**A**) CK group, (**B**) 4000VD group, (**C**) DI group, (**D**) 1000VD+DI group, (**E**) 2000VD+DI group, (**F**) 4000VD+DI group.

As shown in Figure 5, there are only a tiny number of TUNEL-positive cells (brown) in the intestinal villi of the CK group and the 4000VD group, while the positive cells in the DI group are significantly increased. From the 1000VD+DI group to the 4000VD+DI group, the positive cells gradually decreased.

#### 3.4.5. Effect of VD_3_ Addition on the Level of Polysaccharide Secretion in Goblet Cells of Jejunal Tissue

**Figure 6:**
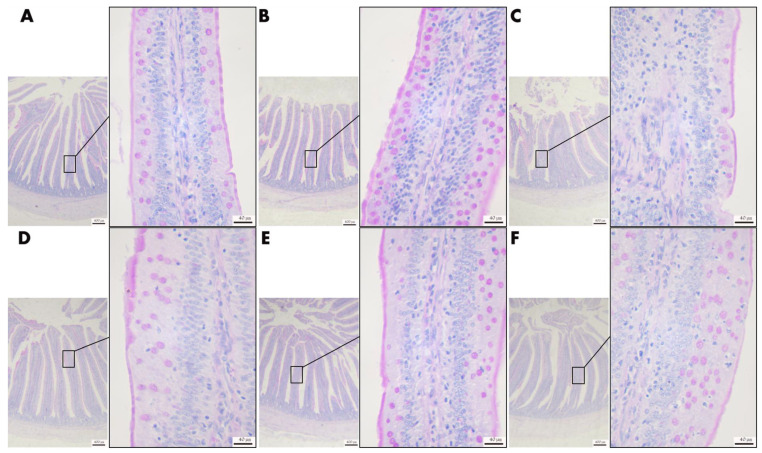
Effects of different levels of VD_3_ supplementation on jejunal polysaccharide secretion in the Wenchang chicken oxidative stress model (40× and 400×). (**A**) CK group, (**B**) 4000VD group, (**C**) DI group, (**D**) 1000VD+DI group, (**E**) 2000VD+DI group, (**F**) 4000VD+DI group.

As shown in Figure 6, the polysaccharide in the jejunal villi in the CK group was colored purple, rich in content, and evenly distributed; the polysaccharide expression in the intestinal villi in the 4000VD group was more densely and evenly distributed. In contrast, the polysaccharide coloration in the DI group was sparse, and the polysaccharide coloration gradually recovered with the increase of VD_3_ addition.

### 3.5. Effect of VD_3_ Addition on the Liver and Kidney Injury

#### 3.5.1. Determination of Biomarkers of Liver and Kidney Injury in Serum

**Figure 7:**
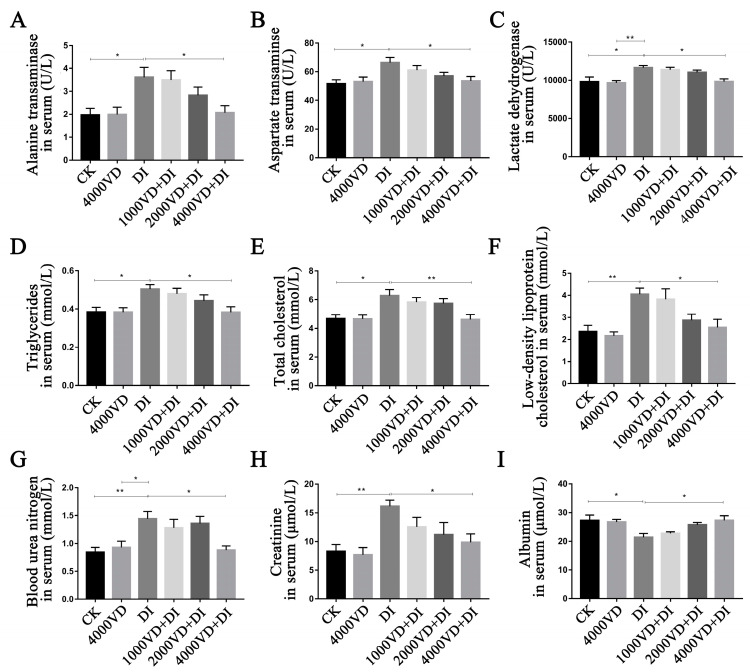
Effects of different levels of VD_3_ supplementation on serum biochemical indexes of the Wenchang chicken oxidative stress model. (**A**) ALT activity in the serum of each group, (**B**) AST activity in the serum of each group, (**C**) LDH activity in the serum of each group, (**D**) TG content in the serum of each group, (**E**) T-CHO content in the serum of each group, (**F**) LDL-C content in the serum of each group, (**G**) BUN content in the serum of each group, (**H**) CR content in the serum of each group, (**I**) ALB content in the serum of each group. (Statistical analysis of the data was performed using the Duncan method to analyze the significance of differences between samples by IBM SPSS software, * *p <* 0.05, ** *p <* 0.01).

As shown in Figure 7, oxidative stress caused an increase in the activity of AST, ALT, and LDH, and the content of BUN, LDL-C, T-CHO, CR, and TG, and a decrease in ALB content. Among them, there were significant differences in AST, ALT, ALB, LDH, TG, and T-CHO between the DI group and the CK group; the 4000VD group (*p <* 0.05), and the CR, LDL-C, and BUN content in the DI group were extremely significantly different from the CK group (*p <* 0.01). With the increase in the concentration of VD_3_, the abnormal biochemical indexes in the serum caused by DQ gradually returned to normal levels, and there was no significant difference between the CK group and the 4000VD+DI group (*p >* 0.05). The result demonstrates the VD_3_ addition reverses the anomaly of AST, ALT, LDH, BUN, LDL-C, T-CHO, CR, TG, and ALB induced by DQ.

#### 3.5.2. Effect of VD_3_ Addition on the Morphology of the Liver Tissues

**Figure 8:**
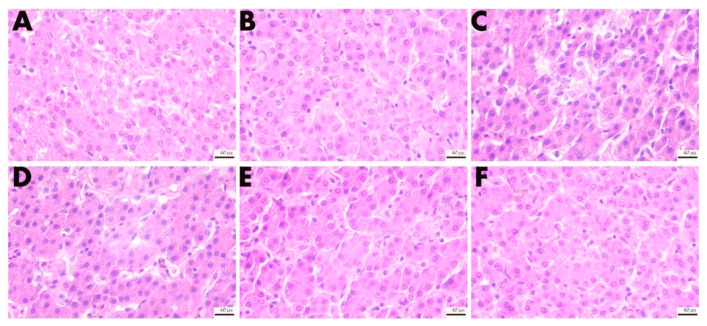
Effects of different levels of VD_3_ supplementation on the morphological structure of liver tissue in the Wenchang chicken oxidative stress model (400×). (**A**) CK group, (**B**) 4000VD group, (**C**) DI group, (**D**) 1000VD+DI group, (**E**) 2000VD+DI group, (**F**) 4000VD+DI group.

As shown in Figure 8, oxidative stress caused the hepatocellular space to increase, the arrangement to be sparse, and nuclear condensation. After treatment with different concentrations of VD_3_, the pathological characteristics were improved; after adding 2000 IU/kg, the arrangement of hepatocytes was gradually clear and orderly, and the nuclear consolidation was reduced. The result indicates that the liver injury was alleviated by VD_3_.

### 3.6. Effect of VD_3_ Addition on the Antioxidant-Related Index

**Figure 9:**
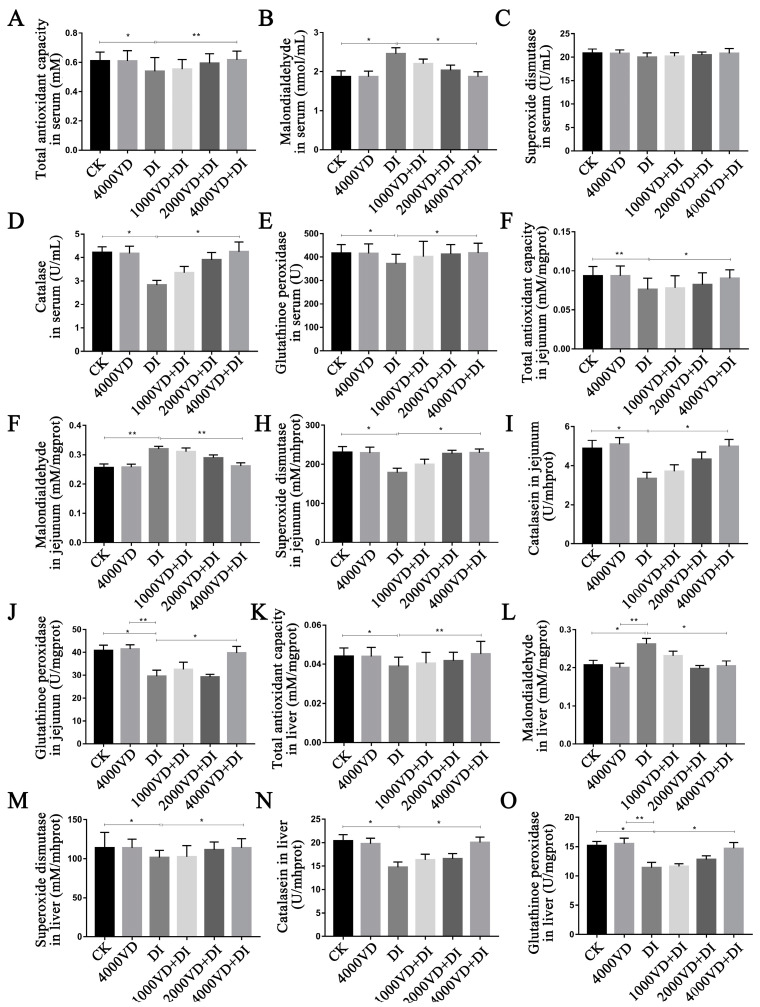
Effects of different supplemental levels of VD_3_ on oxidation-related indexes of the Wenchang chicken oxidative stress model. (**A**–**E**): in serum. (**A**) T-AOC, (**B**) MDA content, (**C**) SOD activity, (**D**) CAT activity, (**E**) GSH-Px activity. (**F**–**J**): in the jejunum. (**F**) T-AOC, (**G**) MDA content, (**H**) SOD activity, (**I**) CAT activity, (**J**) GSH-Px activity. (**K**–**O**): in the liver. (**K**) T-AOC, (**L**) MDA content, (**M**) SOD activity, (**N**) CAT activity, (**O**) GSH-Px activity. (Statistical analysis of the data was performed using the Duncan method to analyze the significance of differences between samples by IBM SPSS software, * *p <* 0.05, ** *p <* 0.01).

As shown in Figure 9, the oxidative stress induced by DQ showed a significant influence on serum, liver, and jejunum antioxidant-related indices. After the injection of DQ, compared with the CK group, the serum, jejunum, and liver T-AOC, CAT and GSH-Px significantly decreased, and the MDA content significantly increased (*p <* 0.05). However, the difference is high in the jejunum T-AOC and MDA (*p <* 0.01). Oxidative stress caused a significant decrease in SOD enzyme activity in the DI group compared with the CK group in the jejunum and liver (*p <* 0.05), and a reduction in serum SOD enzyme activity but no significant difference (*p >* 0.05). As the dosage of VD_3_ gradually increased, the effect of DQ on antioxidant indexes decreased gradually, and there was no significant difference between the 4000 VD+DI group and the CK group (*p >* 0.05). The result suggests that the antioxidant-related index was improved by treatment of VD_3_.

## 4. Discussion

VD is a fat-soluble vitamin commonly used in livestock and poultry production. Studies have shown that VD_3_ plays a significant role in upregulating the expression of certain antioxidant and anti-inflammatory cytokines [31], and can protect animals’ oxidative stress by increasing the Nrf2 expression [32,33]. As an indispensable vitamin for the body, the vitamins have a certain effect on the growth performance of geese, layer chickens or broilers, and other poultry [34,35]. Given the antioxidant properties of vitamins, the role of dietary carotenoids, VD, vitamin E, and vitamin C in biological systems and muscle foods is also worth investigating [36,37,38]. The study suggested that adding 25-OH-D_3_ 69 μg/kg can improve the growth performance and immune parameters of broiler chickens [39]. However, the protective effect of the VD_3_ supplement on growth performance, slaughter performance, organ index, biochemical indicators, and tissue damage of Wenchang chickens is not clear. This study is about the relieving effect of adding different doses of VD_3_ to the Wenchang chicken on the oxidative stress induced by the intraperitoneal injection of DQ. The result displayed that VD_3_ supplementation could improve weight loss in the Wenchang chicken, the dressing percentage, and the pH of the leg muscle after dressing 1 h and 24 h (*p <* 0.05). VD_3_ increased the VH, decreased the CD, and changed the intestinal morphology and liver injury (*p <* 0.05). VD_3_ could promote the development of the immune organ of the spleen under oxidative stress, recover the abnormal serum biochemical index of damaged gut, kidney, and liver, and the antioxidant index of serum, jejunum, and liver (*p <* 0.05). This trial provided some evidence that VD_3_ can relieve the oxidative stress injury of broiler chickens induced by DQ, and established a foundation for searching for an effective nutrition intervention strategy.

VD is closely related to oxidative stress and inflammation, manifested in the relationship between its state in the body and the change of some biomarkers, such as IL, MDA, myeloperoxidase (MPO), and SOD [40]. Some reports have shown that patients with inflammatory bowel disease and colitis-related colon cancer have a significant decrease in VDR protein [41]. The relationship between VDR and immune cells mentioned earlier implies that VDR is a bridge for VD_3_ to stimulate immune responses in immune cells, and it also reflects that VD_3_ may be involved in the immune regulation of inflammatory diseases. Studies have also shown that VD_3_ exerts an immunomodulatory effect by activating VDR to regulate the concentration of pro-inflammatory factors and chemokines [42]. The IL-1β and IL-6 always were produced quickly and were transient after issue injury, which stimulates the host defense, but they became the causes of chronic inflammation when their synthesis was maladjusted [43,44]. In the process of inflammation, TNF-α plays a key role in gut barrier-related disease, and it will be a critical point in intestinal inflammatory [45]. Contrary, IL-10 is an anti-inflammatory factor [46]. Adelani et al. (2020) administered diethylnitrosamine to rats, constructed a model of oxidative stress in rats, and reduced the content of IL-1 β, IL-6, and TNF-α in the serum with oxidative stress by feeding diets containing VD_3_ [47]. The increase of hot stress in proinflammatory factor (TNF-α) was reduced in broiler chickens by dietary 25-OH-D_3_ [48]. This study found that the injection of DQ caused the oxidative stress of the Wenchang chicken to cause the body’s inflammatory response, resulting in an abnormal change in the content of IL-1β, IL-6 and TNF-α, and IL-10 (*p <* 0.05). The VD_3_ addition to the diet could increase IL-10 and decrease IL-1β, IL-6, and TNF-α in serum (*p <* 0.05). The therapeutic effect is enhanced with the increased concentration of VD_3_ added. It is suggested that the regulation of cytokines is the way of inhibiting or alleviating inflammation, even as the potential strategy to reduce intestinal inflammation and permeability.

Intestinal disorders in chickens may be associated with oxidative stress [49]. Studies have shown that oxidative stress causes an increase in the secretion of pro-inflammatory cytokines, thereby increasing intestinal permeability, and resulting in intestinal mucosal damage and intestinal morphology destruction [1,50,51]. It is well established that DAO and D-LA can be used as important indicators for detecting the permeability and integrity of the intestinal mucosa [52]. The intestinal permeability of broiler chickens suffering from hot stress was improved by 25-OH-D_3_ [48]. The experiment showed that the content of DAO and D-LA was increased after injecting DQ (*p <* 0.05), demonstrating that DQ caused intestinal damage. This indicated that the oxidative stress model was successfully constructed. While different doses of VD_3_ were added after the DQ injection, the levels of DAO and D-LA were gradually reduced (*p <* 0.05), suggesting that VD_3_ could reverse the intestinal damage caused by DQ. The integrity of the morphology of intestinal villi is related to the body’s absorption of nutrients, and the VH and the CD can best reflect the state of intestinal villi [53,54]. Both epithelial cells and immune cells in the gastrointestinal tract express receptors for VD and vitamin A. Thus, vitamins play an important role in gastrointestinal regulation, such as fat-soluble vitamins that affect the epithelial integrity of the mucosal barrier and the immune system [55]. In the normal physiological state, the rate of intestinal epithelial cell renewal is in a dynamic balance of apoptosis and proliferation [56]. Its imbalance is one of the important factors leading to damage of the epithelial barrier [57]. In addition to the physical barrier composed of intestinal epithelial cells, the substances secreted by goblet cells are a key component of the intestinal mucus barrier and a line of defense against the entry of microbial pathogenic bacteria and antigens into the intestinal mucosa [58]. In this study, DQ-induced oxidative stress caused a significant decrease in VH and a significant increase in CD. In addition, the positive cell of TUNEL staining was increased, and the purple color of PAS staining was reduced, indicating that DQ caused damage to the intestinal epithelial barrier of the Wenchang chicken. Our study displayed that VD_3_ treatment has an improved effect on the destruction of intestinal villi morphology, the epithelial cell apoptosis, and the reduced polysaccharide secretion in goblet cells, and the better the improvement effect with the gradual increase of the concentration of addition.

Changes in the liver and kidney index suggest possible organ damage from oxidative stress. Injecting DQ can cause severe damage to both organs [3]. There are many detectable biochemical indicators in the body’s serum to assess whether an organ is damaged. The ALT and AST are transaminases synthesized by the liver, and when liver cells are damaged, they are released into the bloodstream [3]. Similarly, LDH, TCHO, and ALB are also widely used to evaluate liver damage [59], BUN and CR are widely used to assess renal function, and TG and LDL-C are biomarkers of lipid metabolism. The liver and kidneys are essential organs that guarantee the synthesis of the active form of VD_3_ [60]. In Adelani’s study, it was noted that feeding a model of oxidative stress constructed from diethylnitrosamine to a diet containing VD_3_ could reduce AST, ALT, and ALB levels in rat serum [47]. In this study, we detected the biochemical damage markers of multiple tissues and organs in the serum, such as ALT, AST, ALB, LDH, BUN, LOL-C, TCHO, CR, and TG. It was found serum biochemical indicators were abnormalities after injecting DQ. Through the treatment of different levels of VD_3_, the abnormal indicators in the serum were restored at different levels. We examined liver tissue lesions and found that oxidative stress induced by DQ caused liver damage. This can be seen by looking at H&E-stained sections. DQ can cause structural disorders in the hepatic lobule and increase the space between hepatocytes. The results of this experiment show that DQ injection causes damage to the intestines, liver, and kidneys, which is consistent with Xing’s study [61]. With different levels of VD_3_ treatment, we can observe that it relieves the tissue structure damage caused by oxidative stress to varying degrees. Therefore, the study demonstrated that VD_3_ intervention could protect against the damage to the intestine, liver, and kidneys induced by DQ.

Studies have shown that VD_3_ may exhibit its antioxidant function by promoting the expression of Klotho and Nrf2 [62,63]. Lin et al. (2022) found that VD_3_ enhances the expressive of HO-1, NQO1, and the level of SOD, GSH, and T-AOC to alleviate the oxidative stress through activating the pathway of PI3K/AKT/Nrf2 in APP/PS1 transgenic mice [64]. The inhibition of enzyme activity (CAT, SOD) in yellow catfish induced by LPS was reversed with the treatment of exogenous VD_3_ [65]. The effect of enhancing the serum T-AOC content and the serum CAT activity is more pronounced when adding 25-OH-D_3_ to the low-calcium phosphorus feed of the broiler body [35]. In this study, testing of antioxidant markers of serum, liver, and jejunal tissue were undertaken. The results showed that the MDA content was increased, whilst SOD, GSH-Px, and CAT were reduced after treating with DQ. The indicator T-AOC, which measures the overall antioxidant capacity, was significantly reduced. Obviously, through different degrees of VD_3_ treatment, we can observe that abnormal antioxidant enzyme activities and MDA are alleviated and reduced, respectively. Specifically, VD_3_ maintains the redox balance in the body and reduces oxidative damage. This study demonstrated that VD_3_ positively affects the activity of antioxidant enzymes.

## 5. Conclusions

In this trial, the result showed that injecting DQ into Wenchang chickens established a model of oxidative stress successfully, and the oxidative stress injury could be alleviated to varying degrees by adding different levels of VD_3_, of which 4000 IU/kg has the best effect. These results indicate that VD_3_ can alleviate the impact of oxidative stress on the performance of livestock when they are subjected to oxidative stress. However, the mechanism is still unclear. This study thus lays a theoretical foundation for the addition of a VD_3_ supplement for the purpose of protecting broiler growth.

## Figures and Tables

**Table 1:** Basal diet composition and its nutrient level (air-dried basal) (%).

Ingredient	1~21 d	22~38 d
Maize	52.50	58.80
Soybean meal	40.00	33.80
Soybean oil	3.00	3.00
Dicalcium phosphate	1.9	1.8
Limstore powder	1.08	1.22
Salt	0.37	0.37
Lysine	0.05	0.03
Methionine	0.19	0.07
Premix ^1^	0.80	0.80
Choline chloride	0.11	0.11
Total	100.00	100.00
Nutritional levels ^2^		
Metabolisable energy (MJ/kg)	12.42	12.62
Crude protein	21.77	19.65
Calcium	1.00	1.02
Available phosphorus	0.44	0.42
Lysine	1.34	1.15
Methionine	0.55	0.40
Cystine	0.40	0.36

^1^ Premix provided the following per kilogram of diet: VA 9000 IU; VD_3_ 3000 IU; VE 26 IU; VK3 1.20 mg; VB1 3.00 mg; VB2 8.00 mg; VB6 4.40 mg; VB12 0.012 mg; niacin 45 mg; calcium pantothenate 15 mg; folic acid 0.75 mg; biotin 0.20 mg; choline chloride 1100 mg; Fe 100 mg; Cu 10 mg; Zn 108 mg; Mn 120 mg; I 1.5 mg; and Se 0.35 mg. ^2^ CP was the measured value, while the others were all calculated values.

**Table 2:** Determination of the growth performance of the Wenchang chicken.

Item	Initial Weight (g)	Final Weight (g)	ADG (g)	ADFI (g)	F/G
CK	109.33 ± 4.56 ^a^	565.84 ± 34.18 ^a^	16.30 ± 1.09 ^a^	30.29 ± 1.62 ^bc^	1.87 ± 0.18 ^a^
4000VD	109.06 ± 5.07 ^a^	566.58 ± 47.79 ^a^	16.34 ± 1.47 ^a^	30.44 ± 1.50 ^bc^	1.87 ± 0.16 ^a^
DI	109.01 ± 6.90 ^a^	546.61 ± 38.87 ^c^	15.62 ± 1.10 ^a^	29.89 ± 1.82 ^c^	1.92 ± 0.19 ^a^
1000VD+DI	108.39 ± 5.56 ^a^	547.04 ± 36.44 ^c^	15.66 ± 1.07 ^a^	29.51 ± 2.00 ^c^	1.89 ± 0.19 ^a^
2000VD+DI	110.22 ± 6.58 ^a^	552.50 ± 38.27 ^bc^	15.80 ± 1.17 ^a^	28.75 ± 2.21 ^c^	1.82 ± 0.17 ^a^
4000VD+DI	109.52 ± 6.79 ^a^	564.41 ± 36.73 ^ab^	16.25 ± 1.12 ^a^	31.83 ± 1.71 ^a^	1.96 ± 0.13 ^a^
*F*	0.828	4.766	0.808	3.176	0.759
*P*	0.530	<0.001	0.549	0.014	0.584

Statistical analysis of the data was performed using the Duncan method to analyze the significance of differences between samples by IBM SPSS software. Means with similar superscripts in the same column indicate no significant differences (*p >* 0.05), and those with different superscripts in the same column indicate significant differences (*p <* 0.05).

**Table 3:** Slaughter performance of the Wenchang chicken (%).

Item	Dressing Percentage	Percentage of Eviscerated Yield
CK	92.66 ± 1.07 ^a^	77.39 ± 1.12 ^a^
4000VD	92.68 ± 1.50 ^a^	77.27 ± 1.23 ^a^
DI	91.66 ± 0.80 ^b^	76.37 ± 1.24 ^b^
1000VD+DI	91.91 ± 1.08 ^ab^	76.94 ± 1.28 ^ab^
2000VD+DI	92.49 ± 0.96 ^a^	77.07 ± 1.22 ^ab^
4000VD+DI	92.27 ± 1.11 ^ab^	76.94 ± 1.05 ^ab^
*F*	2.55	2.113
*P*	0.032	0.070

Statistical analysis of the data was performed using the Duncan method to analyze the significance of differences between samples by IBM SPSS software. Means with similar superscripts in the same column indicate no significant differences (*p >* 0.05), and those with different superscripts in the same column indicate significant differences (*p <* 0.05).

**Table 4:** Organ index of the Wenchang chicken (mg/g).

Item	Liver Index	Bursa of Fabricius Index	Kidney Index	Spleen Index
CK	22.70 ± 0.68 ^a^	4.00 ± 0.24 ^a^	2.41 ± 0.06 ^a^	2.69 ± 0.22 ^a^
4000VD	22.73 ± 0.57 ^a^	4.02 ± 0.16 ^a^	2.43 ± 0.07 ^a^	2.67 ± 0.14 ^a^
DI	21.39 ± 0.43 ^a^	3.48 ± 0.15 ^a^	2.19 ± 0.07 ^b^	2.09 ± 0.16 ^b^
1000VD+DI	21.72 ± 0.45 ^a^	3.57 ± 0.25 ^a^	2.29 ± 0.08 ^ab^	2.15 ± 0.14 ^b^
2000VD+DI	21.92 ± 0.46 ^a^	3.83 ± 0.10 ^a^	2.34 ± 0.06 ^ab^	2.17 ± 0.14 ^b^
4000VD+DI	22.07 ± 0.67 ^a^	3.87 ± 0.16 ^a^	2.42 ± 0.07 ^a^	2.28 ± 0.13 ^ab^
*F*	0.973	1.287	1.812	2.709
*P*	0.44	0.279	0.119	0.027

Statistical analysis of the data was performed using the Duncan method to analyze the significance of differences between samples by IBM SPSS software. Means with similar superscripts in the same column indicate no significant differences (*p* > 0.05), and those with different superscripts in the same column indicate significant differences (*p* < 0.05).

**Table 5:** Quality identification of the Wenchang chicken pectoral muscle.

Item	pH 1 h	pH 24 h	Cooking Loss	Drip Loss
CK	6.68 ± 0.17 ^a^	5.78 ± 0.04 ^a^	10.91 ± 0.49 ^a^	5.91 ± 1.01 ^a^
4000VD	6.64 ± 0.18 ^ab^	5.85 ± 0.02 ^a^	10.41 ± 0.77 ^a^	5.49 ± 1.76 ^a^
DI	6.55 ± 0.12 ^b^	5.76 ± 0.03 ^a^	12.01 ± 0.76 ^a^	8.13 ± 1.08 ^a^
1000VD+DI	6.54 ± 0.12 ^b^	5.80 ± 0.03 ^a^	12.46 ± 0.58 ^a^	7.89 ± 1.13 ^a^
2000VD+DI	6.56 ± 0.07 ^b^	5.76 ± 0.03 ^a^	12.54 ± 0.36 ^a^	6.35 ± 1.25 ^a^
4000VD+DI	6.58 ± 0.13 ^ab^	5.82 ± 0.05 ^a^	10.84 ± 0.98 ^a^	6.12 ± 0.50 ^a^
*F*	2.334	0.966	1.73	0.45
*P*	0.051	0.445	0.141	0.81

Statistical analysis of the data was performed using the Duncan method to analyze the significance of differences between samples by IBM SPSS software. Means with similar superscripts in the same column indicate no significant differences (*p >* 0.05), and those with different superscripts in the same column indicate significant differences (*p <* 0.05).

**Table 6:** Quality identification of the Wenchang chicken leg muscle.

Item	pH 1 h	pH 24 h	Cooking Loss	Drip Loss
CK	6.66 ± 0.13 ^a^	6.28 ± 0.16 ^a^	15.07 ± 1.67 ^a^	5.51 ± 0.38 ^a^
4000VD	6.65 ± 0.13 ^a^	6.29 ± 0.17 ^a^	15.12 ± 1.32 ^a^	5.60 ± 0.52 ^a^
DI	6.54 ± 0.12 ^b^	6.11 ± 0.83 ^b^	16.56 ± 1.10 ^a^	6.61 ± 0.87 ^a^
1000VD+DI	6.56 ± 0.09 ^ab^	6.13 ± 0.15 ^b^	16.39 ± 0.74 ^a^	6.32 ± 1.09 ^a^
2000VD+DI	6.59 ± 0.12 ^ab^	6.17 ± 0.12 ^ab^	15.68 ± 0.96 ^a^	5.85 ± 0.96 ^a^
4000VD+DI	6.63 ± 0.09 ^ab^	6.21 ± 0.24 ^ab^	15.34 ± 1.14 ^a^	5.81 ± 0.20 ^a^
*F*	2.461	3.023	0.274	0.173
*P*	0.041	0.016	0.925	0.97

Statistical analysis of the data was performed using the Duncan method to analyze the significance of differences between samples by IBM SPSS software. Means with similar superscripts in the same column indicate no significant differences (*p >* 0.05), and those with different superscripts in the same column indicate significant differences (*p <* 0.05).

## Data Availability

All the data from this study are included in the article, and the corresponding authors can be contacted directly for further queries.
